# *Coxiella burnetii* Phagocytosis Is Regulated by GTPases of the Rho Family and the RhoA Effectors mDia1 and ROCK

**DOI:** 10.1371/journal.pone.0145211

**Published:** 2015-12-16

**Authors:** Romina P. Salinas, Rodolfo M. Ortiz Flores, Jesús S. Distel, Milton O. Aguilera, María I. Colombo, Walter Berón

**Affiliations:** Instituto de Histología y Embriología, Facultad de Ciencias Médicas, Universidad Nacional de Cuyo—CONICET, Mendoza, 5500, Argentina; University of Arkansas for Medical Sciences, UNITED STATES

## Abstract

The GTPases belonging to the Rho family control the actin cytoskeleton rearrangements needed for particle internalization during phagocytosis. ROCK and mDia1 are downstream effectors of RhoA, a GTPase involved in that process. *Coxiella burnetii*, the etiologic agent of Q fever, is internalized by the host´s cells in an actin-dependent manner. Nevertheless, the molecular mechanism involved in this process has been poorly characterized. This work analyzes the role of different GTPases of the Rho family and some downstream effectors in the internalization of *C*. *burnetii* by phagocytic and non-phagocytic cells. The internalization of *C*. *burnetii* into HeLa and RAW cells was significantly inhibited when the cells were treated with *Clostridium difficile* Toxin B which irreversibly inactivates members of the Rho family. In addition, the internalization was reduced in HeLa cells that overexpressed the dominant negative mutants of RhoA, Rac1 or Cdc42 or that were knocked down for the Rho GTPases. The pharmacological inhibition or the knocking down of ROCK diminished bacterium internalization. Moreover, *C*. *burnetii* was less efficiently internalized in HeLa cells overexpressing mDia1-N1, a dominant negative mutant of mDia1, while the overexpression of the constitutively active mutant mDia1-ΔN3 increased bacteria uptake. Interestingly, when HeLa and RAW cells were infected, RhoA, Rac1 and mDia1 were recruited to membrane cell fractions. Our results suggest that the GTPases of the Rho family play an important role in *C*. *burnetii* phagocytosis in both HeLa and RAW cells. Additionally, we present evidence that ROCK and mDia1, which are downstream effectors of RhoA, are involved in that process.

## Introduction

The dynamic remodeling of actin cytoskeleton is intimately involved in essential cellular processes such as cell adhesion and motility [1], apoptosis [2], endocytosis and phagocytosis [3].

The small GTPases of the Rho family regulate a wide range of cellular activities including cell cycle, morphogenesis, gene transcription, cell adhesion and motility, and vesicular trafficking [4–6]. Some of these functions are tightly associated with the actin cytoskeleton dynamics. The best characterized members of the Rho family are RhoA, Rac1, and Cdc42 which, during cell motility, regulate the formation of stress fibers, lamellipodia and filopodia, respectively, [7,8]. Rho GTPases and the actin cytoskeleton are known to be involved in macropinocytosis and clathrin-dependent and independent endocytosis [3,9,10], and also in endoplasmic reticulum (ER)-Golgi transport during cell secretion [3,11,12].

To form actin filaments, actin nucleation requires factors that can be classified into three groups: the Arp2/3 complex and its nucleation promoting factors, formins, and the tandem-monomer-binding nucleators [13]. The factors mDia1 and mDia2 are members of a subset of formins known as Diaphanous-related formins (Drfs), which have the ability to nucleate and polymerize linear actin filaments [14,15]. At the plasma membrane, both mDia1 and mDia2 can form lamellipodia and filopodia [16,17]. Within the cytoplasm, mDia1 gives rise to stress fibers [18,19] and mDia2 controls the actin dynamics that contributes to vesicle movement [20]. The factors mDia1 and mDia2 have been demonstrated to be involved in actin dynamics leading to the formation of the phagocytic cup in macrophages [21]. In particular, mDia binds directly to both profilin and RhoA, which are recruited around phagocytic cups that are induced by fibronectin-coated beads, suggesting that RhoA regulates actin polymerization by targeting profilin through p140mDia beneath the specific region of plasma membranes [22]. In addition, the interaction of IQGAP with mDia1 is required for phagocytosis and phagocytic cup formation. Moreover, IQGAP mediates the localization of the actin filament nucleator mDia1 [23].

The three Rho isoforms (A, B and C) have several common effectors such as mDia and Rho-kinases (ROCK) 1 and 2, which are both essential for stress fibers formation and focal adhesion organization during cell movement [24,25]. The activation of Rho-kinase also modulates contractile ring formation during cytokinesis [26]. ROCK1 appears to be essential for the formation of stress fibers, whereas ROCK2 appears to be necessary for phagocytosis and cell contraction, both of which are dependent on the phosphorylation of the myosin light chain (MLC) and the MLC phosphatase [27–29].

Phagocytosis is the process developed by cells to engulf particulate material such as apoptotic cells, cell debris and, even, inert particles. Moreover, phagocytosis represents a crucial event that triggers host’s defense mechanisms against invading pathogens. Nevertheless, several pathogens have acquired different strategies to evade these mechanisms to survive and multiply within the host´s cell [30,31]. The phagocytic process is initiated by a recognition step in which ligands on the particle surface bind receptors on the membrane of host’s cells [32]. The ligand-receptor interaction leads to actin cytoskeleton and membrane rearrangements that permit, in the first place, particle engulfment and, in the second, particle sequestration into a phagosome which precedes maturation into a phagolysosome [33,34].

The GTPases of the Rho family control the actin cytoskeleton rearrangements needed for particle internalization by the phagocytes [35]. Fcγ and complement receptor-mediated phagocytosis, also termed Type I and Type II phagocytosis respectively, have been described in macrophages. Cdc42 and Rac1 are activated early in FcγR-mediated phagocytosis, mostly at the rims of the cup [36,37]. Firstly, Cdc42 activates and accumulates preferentially in the tips of the extending pseudopodia [37]. Soon afterwards, after Cdc42 activation and during closure, Rac1 is activated and localized throughout the phagocytic cup, while Rac2 is activated later on, mostly at the base of the cup [37,38]. RhoA seems to be primarily involved in CR3-mediated phagocytosis [36,39]. Even though some reports support the hypothesis that RhoA is unnecessary in FcγR-mediated phagocytosis [21,36,40], others suggest otherwise [41,42].

*Coxiella burnetii*, the causative agent of human Q fever, is an obligate intracellular bacterium found in a wide range of hosts, including livestock and humans. In the case of humans, they acquire the primary infection via the inhalation of contaminated aerosols [43,44]. Infected animals excrete *C*. *burnetii* in milk, urine and feces, and the bacteria are dispersed together with the amniotic fluids and the placenta during animal birthing. *C*. *burnetii* can survive long periods in the environment, since it is highly resistant to heat, drying and common disinfectants. *C*. *burnetii* infects mainly monocytes/macrophages, but it can also infect a wide variety of host cells *in vitro* [45]. This bacterium resides in an acidic parasitophorous vacuole (PV), which has late endosome-lysosome characteristics [46–49]. Recently it has been shown that the PV also interacts with the autophagic pathway, acquiring autophagosomal features [46,48,50]. Interestingly, we have shown that the PV biogenesis is regulated by actin and Rho family GTPases [51]. More recently, we have demonstrated that cortactin is involved in *C*. *burnetii* entry into the host´s cells [52].

It has been demonstrated that cytochalasin D inhibits *C*. *burnetii* uptake [53–55] and that the C3 exotransferase of *Clostridium botulinum*, a GTPase Rho inhibitor, inhibits membrane protrusion when the cells are infected with *C*. *burnetii* [56]. Yet, the role that the actin cytoskeleton of the host´s cell plays in the *C*. *burnetii* entry process remains to be fully characterized.

This report describes the involvement of the GTPases of the Rho family, and the effectors ROCK and mDia1 in *C*. *burnetii* internalization into HeLa and RAW cells. We observed that the overexpression of dominant negative mutants of RhoA, Rac1 and Cdc42 in HeLa cells inhibited bacterium uptake, thereby suggesting that these three GTPases are important for internalization. Similar results were obtained when specific siRNA to RhoA and Rac1 were used. We also analyzed the role of ROCK in internalization using a specific inhibitor, and by silencing the protein with a siRNA. Both treatments diminished *C*. *burnetii* internalization. Furthermore, we studied the participation of mDia1 in that process and found that the overexpression of a negative mutant of mDia1 reduced *C*. *burnetii* uptake. In summary, our results indicate that the GTPases of the Rho family and the RhoA effectors mDia1 and ROCK regulate the internalization of *C*. *burnetii*.

## Materials and Methods

### Materials

Dulbecco’s Modified Eagle's Medium (D-MEM), fetal bovine serum (FBS), penicillin and streptomycin were obtained from Gibco BRL/Life Technologies (Buenos Aires, Argentina). Plasmids encoding EGFP-Rac1, -Cdc42 and -RhoA dominant negative mutants were kindly provided by Dr Philippe Chavrier (Centre National de la Recherche Scientifique/Institut Curie, Paris, France) and Mark R. Phillips (Laboratory of Molecular Rheumatology, NYU, School of Medicine, USA). Plasmids encoding EGFP-mDia1 WT, and the truncated forms -mDia1-ΔN3 and -mDia1-N1 were kindly provided by Dr. Fernandez-Borja (The Netherlands Cancer Institute, Division of Tumor Biology, The Netherlands). Small interfering RNAs (siRNAs) against RhoA (1129127), Rock1 (1130663) and Rac1 (1126011) were purchased from Bioneer (Alameda, USA). The monoclonal anti-RhoA antibody was purchased from Santa Cruz Biotechnology (California, USA); and the monoclonal anti-Rac1 antibody, the monoclonal anti-mDia1 and anti-actin Ab-5 were purchased from BD (Buenos Aires, Argentina). The monoclonal anti-E cadherin antibody (Cell Signaling Technology) was a gift of Dr. Ciocca (Laboratorio de Oncología, IMBECU-CONICET, Mendoza, Argentina). Secondary antibodies were purchased from Jackson ImmunoResearch Laboratories, Inc. (West Grove, PA, USA The rabbit polyclonal anti-*Coxiella* antiserum was kindly provided by Dr Robert Heinzen (Rocky Mountain Laboratories, NIAID, NIH, Hamilton, MT, USA). Toxin B, from *Clostridium difficile*, and the inhibitor Y27632 were from Merck-Calbiochem® (Buenos Aires, Argentina). Phalloidin-FITC and the protease inhibitor cocktail were from Sigma-Aldrich (Buenos Aires, Argentina).

### Cell culture

HeLa cells (Asociación Banco Argentino de Células, Buenos Aires, Argentina) were grown in DMEM supplemented with 10% heat-inactivated FBS, 2.2 g/l sodium bicarbonate, 2 mM glutamine, 100 IU/ml penicillin and 100 μg/ml streptomycin, pH 7, at 37°C under a 5% CO_2_ atmosphere. RAW cells were grown in RPMI supplemented with 10% heat-inactivated FBS, 2.2 g/l sodium bicarbonate, 2 mM glutamine, 100 IU/ml penicillin and 100 μg/ml streptomycin, pH 7, at 37°C under a 5% CO_2_ atmosphere.

### Propagation of phase II *C*. *burnetii*

Clone 4 phase II Nine Mile strain of *C*. *burnetii* which are infective for cells in culture but not for mammals, were provided by Ted Hackstadt (Rocky Mountain Laboratories, NIAID, NIH, Hamilton, MT, USA) and handled in a biosafety level II facility. Non-confluent Vero cells were cultured in T25 flasks at 37°C under a 5% CO_2_ atmosphere in DMEM supplemented with 5% FBS, 0.22 g/l sodium bicarbonate and 20 mM Hepes, pH 7 (MfbH). Cultures were infected with *C*. *burnetii* phase II suspensions for 6 days at 37°C under a 5% CO_2_ atmosphere. In order to prepare cell lysates, cells were frozen at -70°C, then thawed at 37°C, scraped and passed 20 times through a 27-gauge needle connected to a syringe. Cell lysates were centrifuged at 800 x *g* for 10 min at 4°C. Supernatants were centrifuged at 24,000 x *g* for 30 min at 4°C, and pellets containing *C*. *burnetii* were resuspended in phosphate-buffered saline (PBS; 10 mM sodium phosphate, 0.9% NaCl), aliquoted and frozen at -70°C.

### Infection of HeLa and RAW cells with *C*. *burnetii*

Cells (5 x 10^5^) were seeded on sterile glass coverslips placed in 24-well plates and grown overnight in MfbH medium. For infection, a 5 μl aliquot of *C*. *burnetii* suspension was added per well (Multiplicity of infection: 20–40). Cells were incubated for different lengths of time at 37°C under a 5% CO_2_ atmosphere. Cells were fixed and processed for indirect immunofluorescence.

### Subcellular fractionation

HeLa cells were cultured in 60-mm dishes and infected as described above for different lengths of time. Cells were washed with cold PBS and homogenization buffer HB (250 mM sucrose, 3 mM imidazole, pH 7.4), and scraped in HB containing protease inhibitors and 0.5 mM EDTA. Scraped cells were homogenized using a Dounce type homogenizer with a Teflon pestle. The homogenate was centrifuged at 13000 x*g* for 15 sec at 4°C. An aliquot of the supernatant (postnuclear supernatant) was frozen at -20°C (total fraction: T) and the rest was centrifuged at 100,000 x *g* for 30 min at 4°C. The supernatant obtained and the pellet represented the cytosolic (C) and membrane (M) fractions, respectively. Both fractions were analyzed by SDS-PAGE and Western blot.

### Immunofluorescence

Cells were fixed with 2% paraformaldehyde in PBS for 10 min at 37°C, washed with PBS and blocked with 50 mM NH_4_Cl in PBS. After washing, cells were incubated with a rabbit antiserum against *C*. *burnetii* (1:1000) and a donkey anti-rabbit secondary antiserum conjugated to Cy5 diluted 1:600 in PBS containing 0.5% BSA (non-permeabilizing conditions). In this condition, only extracellular bacteria were stained in white pseudo color. After washing, cells were incubated with the same rabbit antiserum against *C*. *burnetii* (1:1000) and a donkey anti-rabbit secondary antibody conjugated to Cy3 diluted 1:600 in PBS containing 0.5% BSA and 0.05% saponin (permeabilizing conditions). Under these conditions, the white-labeled extracellular bacteria were stained in red pseudo color, thus the extracellular one acquired both white and red pseudo colors, while the intracellular ones were only stained in red pseudo color. F-actin was stained with phalloidin-FITC. Coverslips were mounted with Mowiol (Sigma-Aldrich, Buenos Aires, Argentina) and examined under fluorescence microscopy (FV1000 Olympus Confocal Microscope and the FV 10-ASW 1.7 software, Olympus, Japan).

### Cell transfection

HeLa cells were transfected for 6 h with 2 μg/ml pEGFP empty vector or pEGFP plasmids expressing the fusion proteins of EGFP with the dominant negative mutants RhoA N19, Rac1 N17 or Cdc42 N17, mDia1 WT or the truncated forms mDia1-ΔN3 and mDia1-N1. Cell transfection was carried out using Lipofectamine® 2000 (Invitrogen, Buenos Aires, Argentina), according to the manufacturer’s instructions. After 6 h of transfection, cells were washed and incubated for 18 h in MfbH medium at 37°C under a 5% CO_2_ atmosphere The siRNA transfection was performed employing Lipofectamine® 2000 according to the manufacturer’s instructions (Bioneer, Alameda, USA).

### Western blotting

HeLa and RAW cells were cultured in 60 mm dishes and infected as described earlier for different lengths of time. After infection, cells were washed with PBS, scraped into ice-cold lysis buffer (50 mM Tris-HCl, pH 7.2, 1% Triton X-100, 0.5% deoxycholate, 0.1% SDS, 50 mM NaCl, 10 mM MgCl_2_, 2 mM Na_3_VO_4_, 10 mM NaF, 0.5 mg/ml DTT, 2 mM EDTA) supplemented with a protease inhibitor cocktail and kept on ice for 20 min. Lysates were clarified by centrifugation at 2000 x *g* for 15 min at 4°C. Clarified lysates were transferred to clean tubes, mixed with Laemmli buffer and boiled for 5 min. Samples were resolved by SDS-PAGE and the proteins transferred to nitrocellulose membranes using standard procedures. Membranes were blocked for 2 h at 4°C in Tween-Tris-buffered saline (TTBS; 0.1% Tween 20, 100 mM Tris/HCl, 0.9% NaCl) supplemented with 5% BSA, then incubated overnight at 4°C with the appropriate primary antibodies. Membranes were washed three times with TTBS and then incubated for 2 h at room temperature with appropriate peroxidase-conjugated secondary antibodies. Membranes were washed again with TTBS and developed using the ECL Western blotting system (GE Healthcare) according to the supplier’s recommendations. Anti-actin and anti-E cadherin antibodies were used as loading controls. Band densitometry was carried out using ImageJ software (NIH, USA).

### Fluorescence microscopy

Cells were analyzed by fluorescence microscopy using an FV1000 Olympus Confocal Microscope and the FV 10-ASW 1.7 software (Olympus, Japan). Images were processed using ImageJ software. Cell boundaries were marked with dotted lines in all figures showing experiments performed with transfected cells.

### Statistical analysis

Differences between conditions were tested by one-way analysis of variance (ANOVA) and Dunnett’s *post hoc* tests or Student’s *t* single group test. Differences were considered significant at p < 0.05.

## Results

### *Clostridium difficile* toxin B, an inhibitor of Rho family GTPases, diminishes the internalization of *C*. *burnetii* by phagocytic and non-phagocytic cells

It is well known that to accomplish internalization into the host´s cells, several pathogens modulate the GTPases of the Rho family [57]. In previous works, we have demonstrated that actin and Rho GTPases are involved in the intracellular trafficking of *C*. *burnetii* [51]. However, comprehensive studies regarding the role of these GTPases in *C*. *burnetii* entry into host cells are scarce. *Clostridium difficile* toxin B is a pharmacological tool used to study Rho GTPases function. This toxin is a protein that monoglucosylates RhoA, Rac1 and Cdc42, leading to their irreversible inactivation [58,59].

**Fig 1:**
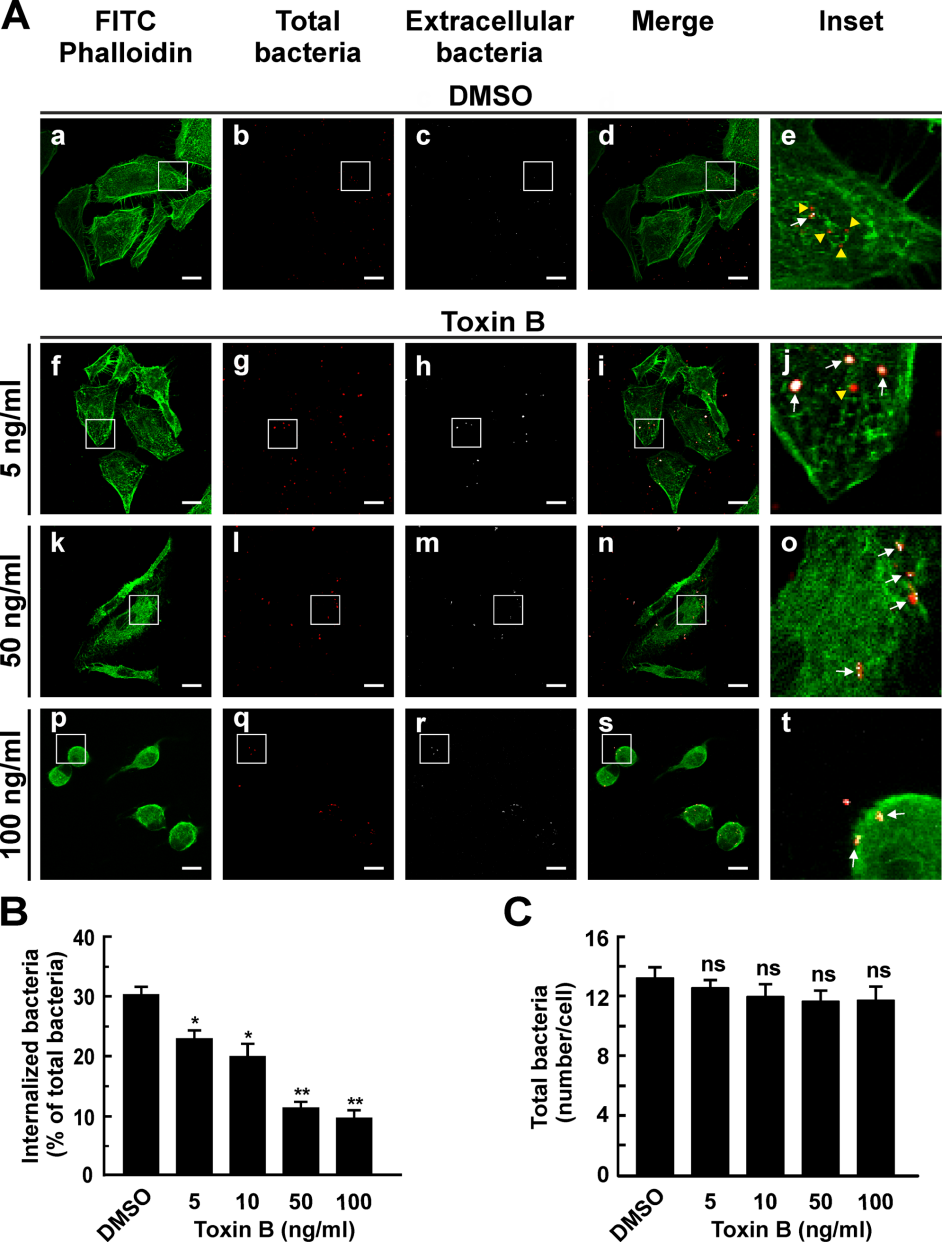
*Clostridium difficile* toxin B diminishes the internalization of *C*. *burnetii* by HeLa cells. (A) HeLa cells were infected with *C*. *burnetii* for 4 h at 37°C in the presence of 0.05% DMSO (control, panels a-e) or with different concentrations of *Clostridium difficile* toxin B (panels f-t). Cells were fixed and processed for indirect immunofluorescence to determine *C*. *burnetii* internalization and F-actin distribution as described in Materials and Methods. Cells were analyzed by confocal microscopy. Micrographs of representative cells are shown. Cells were incubated sequentially with an antibody against *C*. *burnetii* and an appropriate secondary antibody conjugated to Cy5 (white pseudo color) under non-permeabilizing conditions. Under this condition, extracellular bacteria were stained in white pseudo color (panels c, h, m, and r). Then, cells were re-incubated with the same anti-*C*. *burnetii* antibody and an appropriate secondary antibody conjugated to Cy3 (red pseudo color) under permeabilizing conditions. Under this condition total bacteria were stained in red pseudo color (panels b, g, l, and q). In the merged images (panels d, i, n and s) and the insets of the merged images (panels e, j, o, and t), extracellular *C*. *burnetii* is shown in white and red pseudo colors (arrows), while intracellular *C*. *burnetii* is shown in red pseudo color (yellow arrowheads). F-actin was labeled with phalloidin-FITC (green, panels a, f, k, and p). Bars scale: 5 μm. (B) Quantification of *C*. *burnetii* internalized in control and toxin-treated HeLa cells. (C) Quantification of total *C*. *burnetii* associated to control and treated HeLa cells. Between 100 and 120 cells and 1200 and 1600 bacteria were counted in each experiment. Results are expressed as means ± SE of three independent experiments. *p < 0.05, **p < 0.01 compared to the DMSO treatment (one-way ANOVA and Dunnett's *post hoc* test). ns: non-significant differences between groups (p > 0.05).

**Fig 2:**
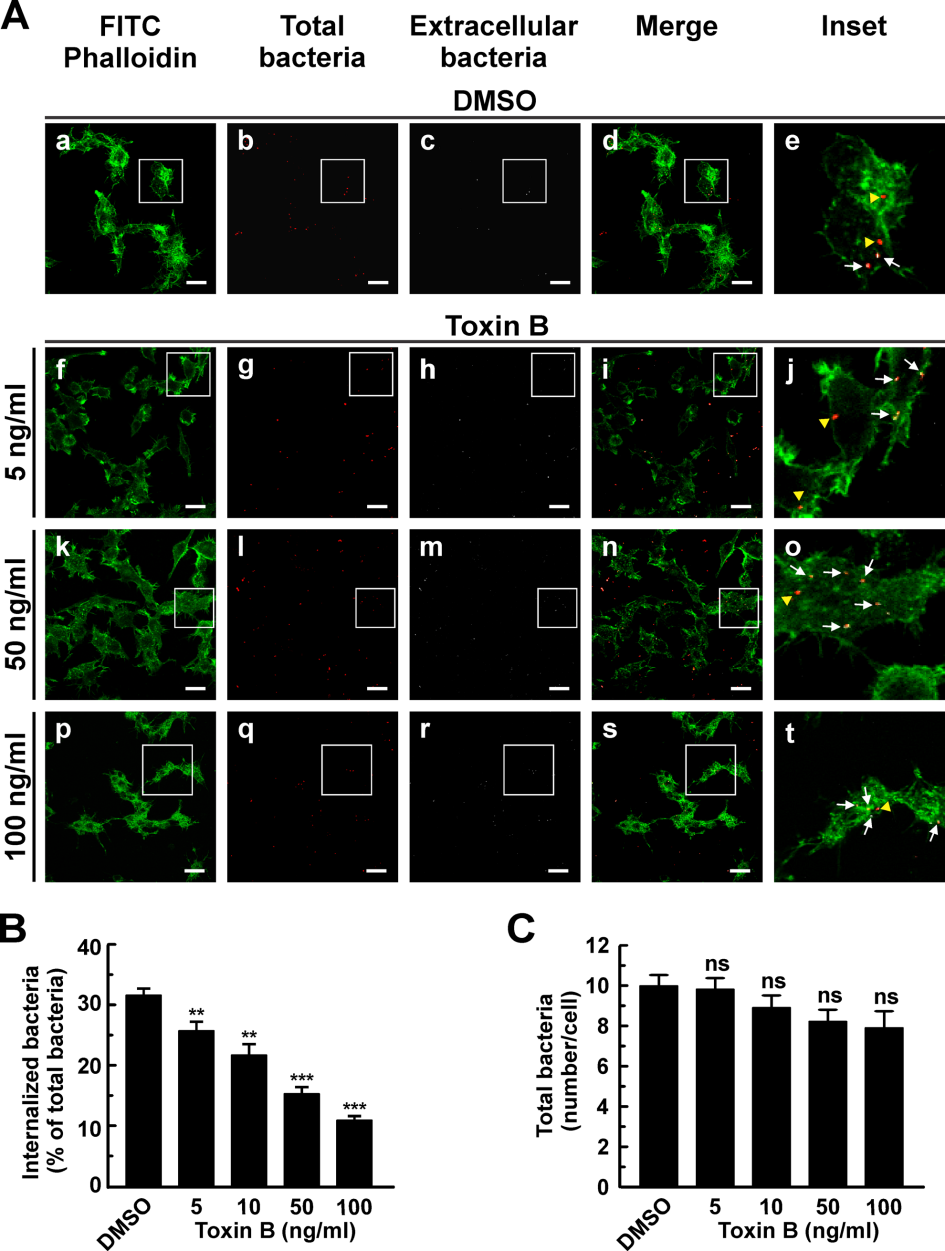
*Clostridium difficile* toxin B diminishes internalization of *C*. *burnetii* by RAW macrophages. (A) RAW cells were infected with *C*. *burnetii* for 4 h at 37°C in the presence of 0.05% DMSO (control, panels a-e) or different concentrations of *Clostridium difficile* toxin B (panels f-t). Cells were fixed and processed for indirect immunofluorescence to determine *C*. *burnetii* internalization and F-actin distribution as described in Materials and Methods. Cells were analyzed by confocal microscopy. Micrographs of representative cells are shown. As indicated in Fig 1, extracellular and total bacteria were stained in white pseudo color (panels c, h, m, and r) and red pseudo color (panels b, g, l, and q), respectively. In the merged images (panels d, i, n, and s) and the insets of the merged images (panels e, j, o, and t), extracellular *C*. *burnetii* is shown in white and red pseudo colors (arrows), while intracellular *C*. *burnetii* is shown in red pseudo color (yellow arrowheads). F-actin was labeled with phalloidin-FITC (green). Bar scale: 10 μm. (B) Quantification of *C*. *burnetii* internalized in control and toxin-treated RAW cells. (C) Quantification of total *C*. *burnetii* associated to control or toxin-treated cells. Between 100 and 120 cells and 1200 and 1600 bacteria were counted in each experiment. Results are expressed as means ± SE of three independent experiments. **p < 0.01, ***p < 0.001, compared to DMSO treatment (one-way ANOVA and Dunnett's *post hoc* test). ns: non-significant differences between groups (p > 0.05).

To determine the role of Rho GTPases in the internalization of *C*. *burnetii*, RAW and HeLa cells (phagocytic and non-phagocytic cells, respectively), were infected for 4 h at 37°C in the presence of different toxin B concentrations. Cells were fixed, processed for indirect immunofluorescence and analyzed by confocal microscopy to evaluate cell morphology and the number of intracellular bacteria. F-actin was stained with phalloidin-FITC. Control cells (DMSO-treated) the typical HeLa cell morphology with typical cortical actin, filopodia and stress fibers (Fig 1A, panel a). As expected, the toxin altered HeLa morphology mainly at high concentrations, a condition under which cells became rounded and lost the typical actin structure (Fig 1A, panel p). In the inset panels of Fig 1A, intracellular bacteria are shown in red pseudo color (yellow arrowheads) while the extracellular ones are shown in red and white pseudo colors (arrows). The number of intracellular bacteria was lower in cells treated with toxin B as compared to the control (Fig 1A, insets, and B). Toxin treatment inhibited *C*. *burnetii* internalization in a dose-dependent manner. Similar results were observed when RAW cells were infected (Fig 2). Even though the toxin-treated RAW cells exhibited milder changes in their shapes as compared to non-treated cells (Fig 2A), they lost the typical actin structures and were less efficient in *C*. *burnetii* internalization (Fig 2A, insets, and 2B). Treatment with increasing concentrations of toxin B did not affect significantly the number of total bacteria associated to HeLa (Fig 1C) or RAW (Fig 2C) cell surfaces. These findings would indicate that the progressive inhibition of the bacterium internalization is not due to a defective in bacterial cell adherence.

These results suggest that the GTPases belonging to the Rho family play a role in *C*. *burnetii* entry into both phagocytic and non-phagocytic cells.

### RhoA and Rac1 GTPases are recruited to the membrane fraction obtained from HeLa cells infected with *C*. *burnetii*

It is known that the GTPases of the Rho family regulate the actin cytoskeleton reorganization beneath the plasma membrane of the host´s cells in contact with particles or microorganisms to be engulfed during phagocytosis [60,61]. GTPases cycle between an active state (GTP-bound) and an inactive one (GDP-bound). It is also known that in the GTP-bound form, GTPases are recruited to membranes and initiate intracellular signal cascades that regulate different cell functions [62,63].

**Fig 3:**
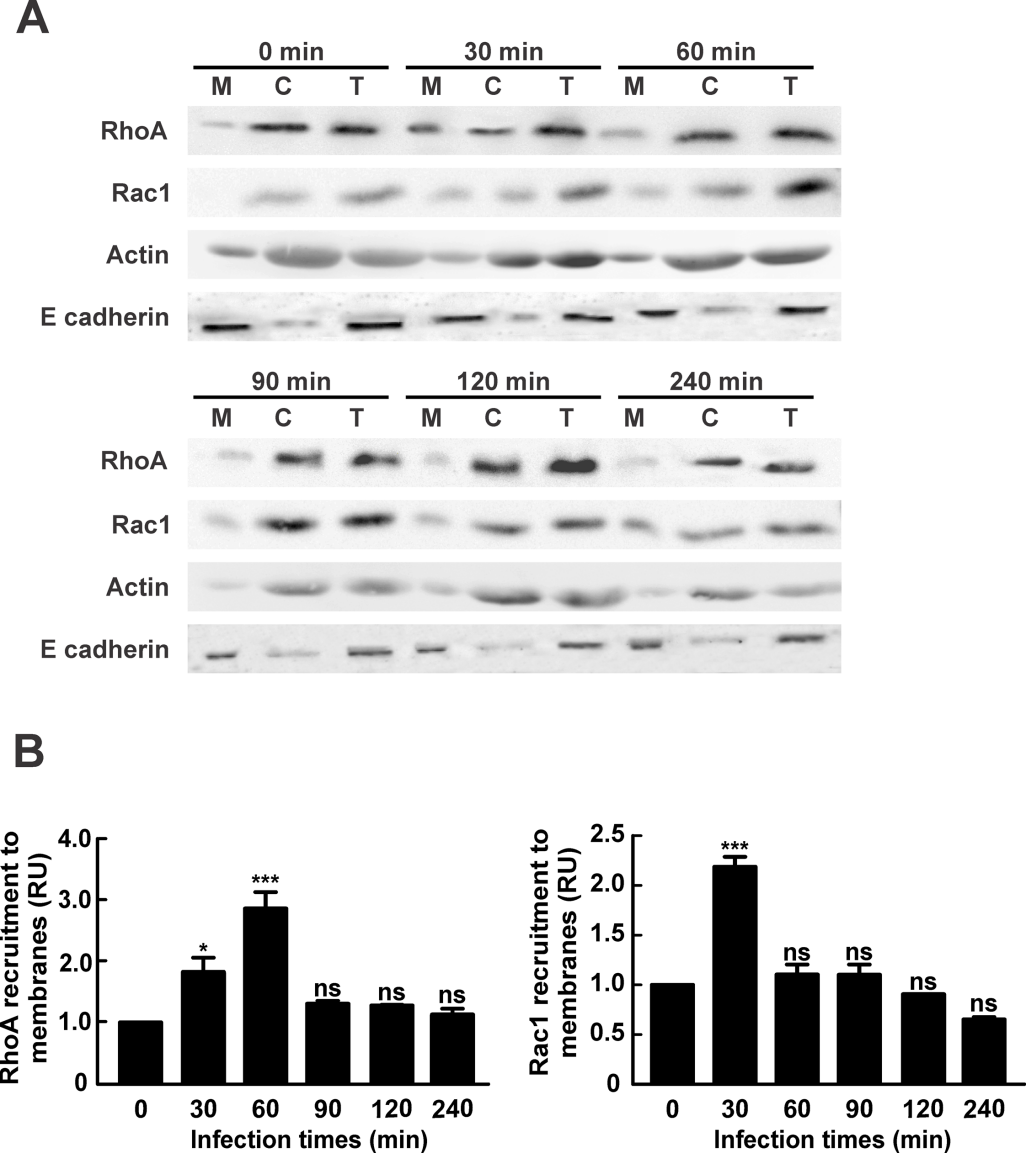
RhoA and Rac1 are recruited to a membrane fraction obtained from cells infected with *C*. *burnetii*. HeLa cells were infected with *C*. *burnetii* for different lengths of time, lysed and centrifuged to obtain postnuclear supernatant, membrane and cytosolic fractions as described in Materials and Methods. (A) Postnuclear supernatant (T: total), cytosol (C) and membrane (M) fractions were analyzed by SDS-PAGE and Western blot using antibodies against RhoA and Rac1. Anti-actin and anti-E cadherin antibodies were used as loading controls. (B) Quantification of RhoA or Rac1 recruitment to the membrane fraction. The band intensities corresponding to RhoA, Rac1, E cadherin and actin were measured by the ImageJ software, and the band intensity ratio between RhoA and E cadherin, and Rac1 and E cadherin in the membrane fractions was calculated. Results are expressed as means ± SE from at least three independent experiments. Mean values were compared with the 0 min infection condition by Student’s *t* test for single group mean (*p < 0.05, ***p < 0.001). ns: non-significant differences between groups (p > 0.05). RU: Relative Units.

We suggest that *C*. *burnetii* estimulates recruitment of Rho GTPases to the host´s cell membrane during phagocytosis. To test our hypotesis, HeLa cells were infected for different periods of time, lysed and cetrifuged to obtain membrane and cytosolic fractions. Postnuclear supernatant (T: total fraction), cytosol (C) and membrane (M) fractions were analyzed by SDS-PAGE and Western blot. Fig 3A and 3B depicts that RhoA recruitment to the membrane fraction increased after 30 min and peaked after 60 min of infection. After this timepoint, the levels of Rho membrane association decreased to basal levels. This result suggests that RhoA is activated during *C*. *burnetii*-host´s cell interaction. Interestingly, when the Rac1 membrane recuitment was analyzed, as shown in Fig 3C and 3D, the maximum level was observed after 30 min of infection, sugesting that Rac1 is also activated, though earlier than RhoA. The membrane recruitment of Cdc42 during infection was also analyzed, but unfortunately the antibody against Cdc42 used was unable to detect the protein even in the total fraction (data not shown). Similar results were obtained in HeLa cells incubated with heat-killed *C*. *burnetii* (S1 Fig). Therefore, the same signaling cascade can be activated by live and heat-killed *C*. *burnetii* during phagocytosis.

### Internalization of *C*. *burnetii* by HeLa cells is inhibited by overexpression of Rho GTPases dominant negative mutants or by silencing these proteins

**Fig 4:**
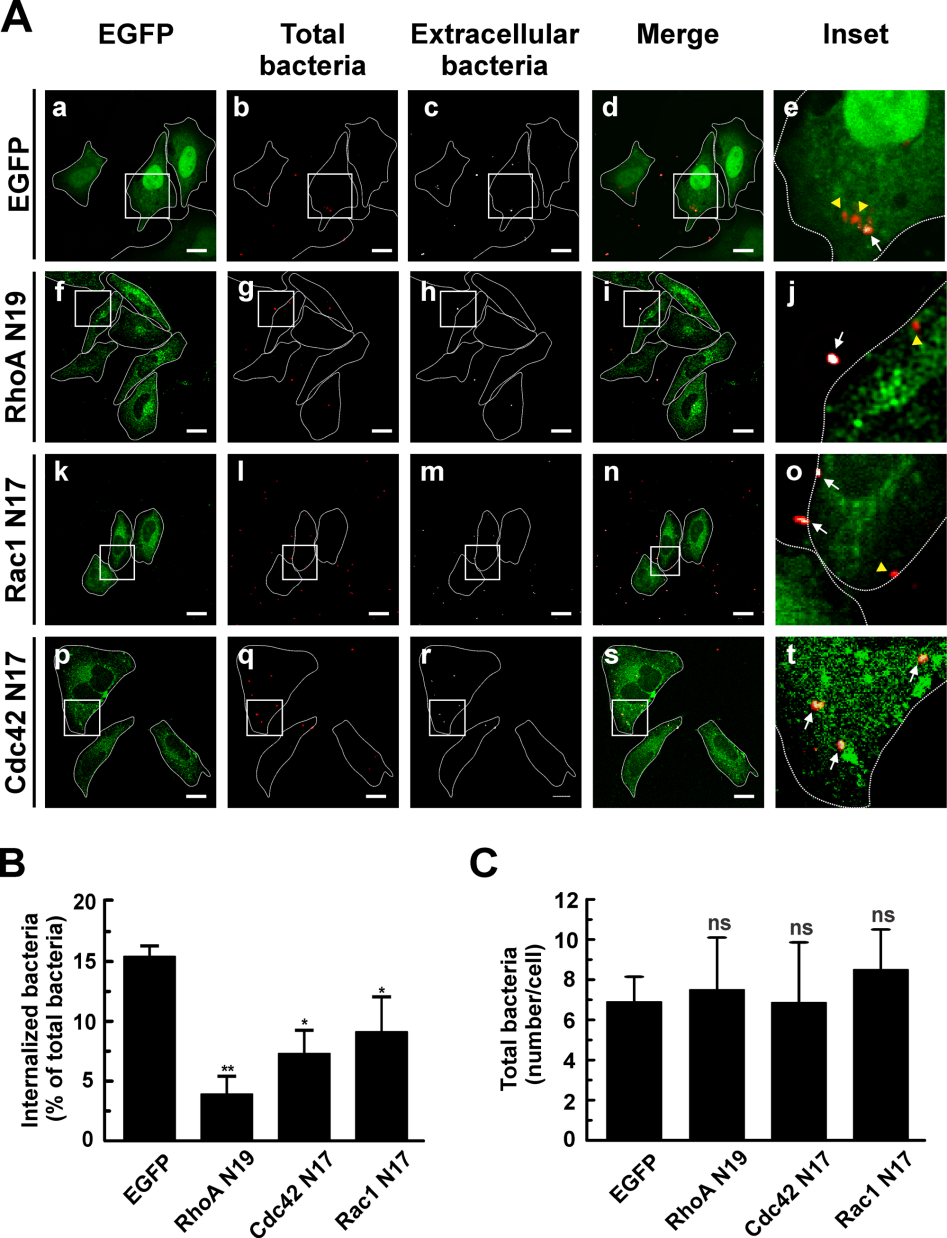
*C*. *burnetti* internalization is inhibited by overexpression of the dominant negative mutants of Rho GTPases. (A) HeLa cells were transfected with pEGFP (panels a-e), pEGFP-RhoA N19 (panels f-j), pEGFP-Cdc42 N17 (panels k-o), or pEGFP-Rac1 N17 (panels p-t). Cells were infected for 4 h at 37°C with *C*. *burnetii* and subsequently fixed and processed for immunofluorescence to determine the levels of *C*. *burnetii* internalization as described in Materials and Methods. Cells were analyzed by confocal microscopy. Representative micrographs are presented. As indicated in Fig 1, extracellular and total bacteria were stained in white pseudo color (panels c, h, m, and r) and red pseudo color (panels b, g, l, and q), respectively. In the merged images (panels d, i, n, and s) and the insets of the merged images (panels e, j, o, and t), extracellular *C*. *burnetii* is shown in white and red pseudo colors (arrows), while intracellular *C*. *burnetii* is shown in red pseudo color (yellow arrowheads). Bars scale: 5 μm. (B) Quantification of *C*. *burnetii* internalized by transfected HeLa cells. (C) Quantification of total *C*. *burnetii* associated to HeLa cells. Between 40 and 60 cells and between 400 and 600 bacteria were counted in each experiment. Results are expressed as means ± SE of three independent experiments. *p < 0.05, **p < 0.01 compared to the EGFP control (one-way ANOVA and Dunnett's *post hoc* test). ns: non-significant differences between groups (p > 0.05).

The inhibitory effect of toxin B in *C*. *burnetii* internalization and recruitment of Rho GTPases to a membrane fraction obtained from infected cells suggests that Rho GTPases are activated during infection. An experimental approach to assess the requirement for the active forms of Rho GTPases during the bacterium entry consists in overexpressing the dominant negative mutants of these proteins. HeLa cells were transfected with pEGFP-RhoA N19, pEGFP-Cdc42 N17 or pEGFP-Rac1 N17 and then infected for 4 h at 37°C. Cells were fixed and processed as mentioned before to evaluate the number of intracellular bacteria. As displayed in Fig 4A, the overexpressed dominant negative mutants featured a diffused distribution in the cytoplasm (Fig 4A, panels f, k and p).

In HeLa cells overexpressing EGFP, a larger number of intracellular bacteria (red pseudo color, yellow arrowheads) was observed in relation to the extracellular ones (red and white pseudo colors, arrows) (Fig 4A, panel e). In HeLa cells overexpressing the dominant negative mutants of the three Rho GTPases, a significant inhibition of *C*. *burnetii* internalization was observed (Fig 4A, panels j, o and t, and B). Total bacteria associated to the cells was not significantly different among the tested constructs (Fig 4C), thus indicating that the low percentage of internalized bacteria is not due to a defective bacterial adherence to cells. The strongest inhibitory effect was observed in cells overexpressing the dominant negative mutant of RhoA (i.e., RhoA N19). These results suggest that the active forms of RhoA, Cdc42 and Rac1 are important for the entry of *C*. *burnetii* into host´s cells.

**Fig 5:**
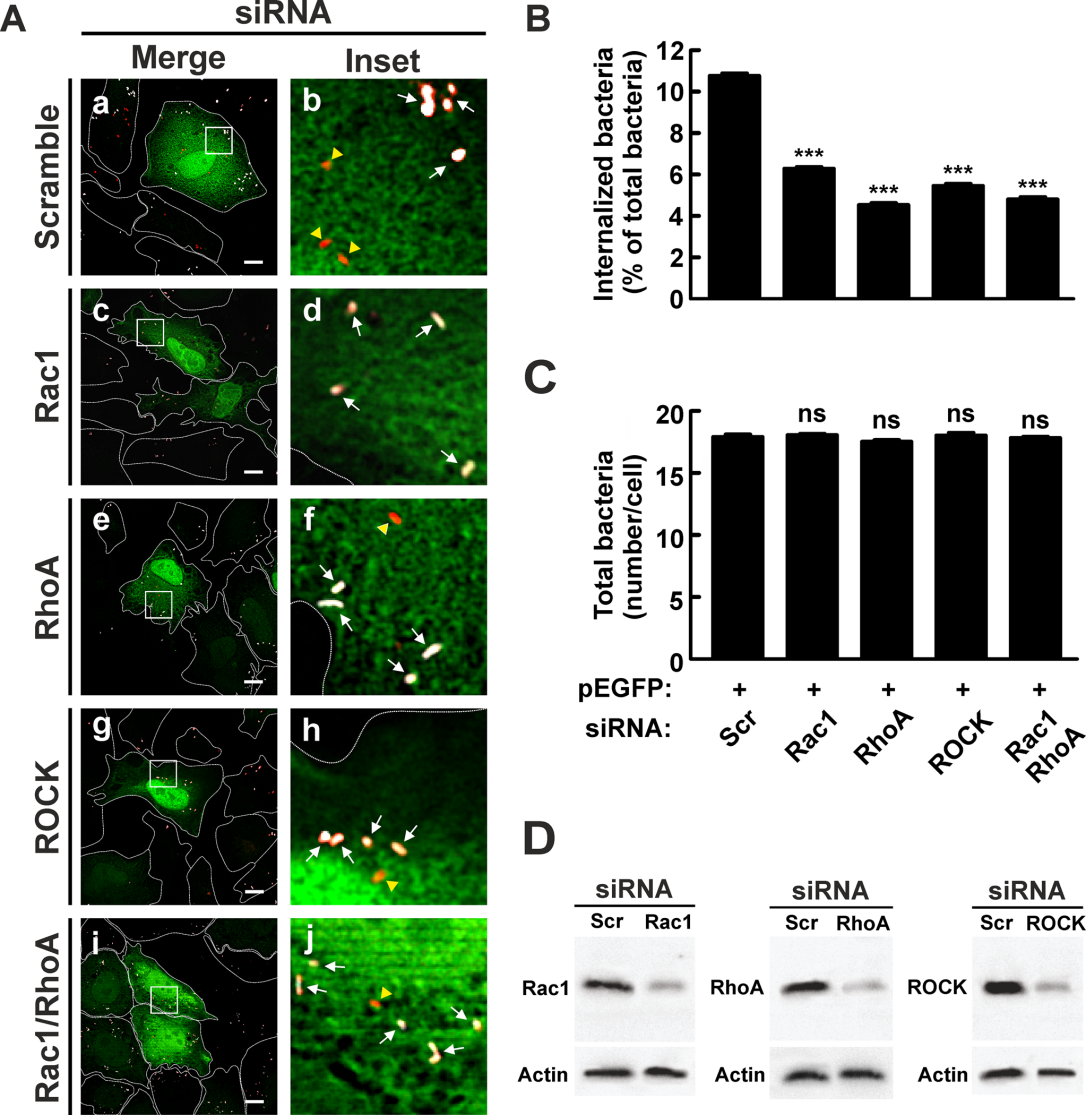
Knockdown of Rho GTPases and Rock inhibits internalization of *C*. *burnetii*. (A) HeLa cells were co-transfected with pEGFP and a scramble (panels a and b), Rac1 (panels c and d), RhoA (panels e and f) or ROCK (panels g and h) siRNAs or the RhoA/Rac1 siRNA combination (panels i and j). Cells were infected for 4 h at 37°C with *C*. *burnetii* and then fixed and processed for immunofluorescence to determine *C*. *burnetii* internalization as described in Materials and Methods. Cells were analyzed by confocal microscopy. Representative micrographs of cells are presented. As indicated in Fig 1, in the merged images (panels a, c, e, g, and i) and the insets of the merged images (panels d, d, f, h, and j), extracellular *C*. *burnetii* is shown in white and red pseudo colors (arrows), while intracellular *C*. *burnetii* is shown in red pseudo color (yellow arrowheads). Scale bar: 5 μm. (B) Quantification of *C*. *burnetii* internalized by transfected HeLa cells. (C) Quantification of total *C*. *burnetii* associated to HeLa cells. Between 40 and 60 cells and between 400 and 600 bacteria were counted in each experiment. Results are expressed as means ± SE of three independent experiments. ***p < 0.001, compared to scramble siRNA (one-way ANOVA and Dunnett's *post hoc* test). ns: non-significant differences between groups (p > 0.05). (D) Lysates of cotransfected HeLa cells were analyzed by SDS-PAGE and Western blot using antibodies against Rac1, RhoA and ROCK. An anti-actin antibody was used as loading control. Scr: scramble siRNA.

To confirm the role of RhoA and Rac1 in the internalization process, these proteins were knocked down by specific siRNAs. HeLa cells were transfected with siRNAs against RhoA and Rac1 and then infected for 4 h at 37°C. Cells were either lysed to analyze the depletion levels of RhoA and Rac1 proteins (see Fig 5D) or fixed and processed as mentioned above in order to evaluate the number of intracellular bacteria. The number of intracellular bacteria (red pseudo color, yellow arrowheads) was lower in HeLa cells transfected with Rac1 (Fig 5A, panels c and d, and B) and RhoA (Fig 5A, panels e and f, and B) siRNAs as compared to that observed in cells transfected with the scramble siRNA (Fig 5A, panels a and b, and B). The depletion of the endogenous RhoA or Rac1 proteins diminished *C*. *burnetii* internalization without significantly affecting the number of total bacteria associated to HeLa cells. This finding suggests that the inhibition of the bacterium internalization is not explained by a defect in bacteria adherence to the cells (Fig 5C). These inhibitory effects are in agreement with those produced by the overexpression of the dominant negative mutants RhoA N19 and Rac1 N17.

To test the possibility of a combined effect that would further inhibit *C*. *burnetii* entry, HeLa cells were cotransfected with siRNAs targeted to RhoA and Rac1 and then infected for 4 h at 37°C. Cells were fixed and processed as described above to evaluate the number of intracellular bacteria. The level of *C*. *burnetii* internalization in cells depleted for both RhoA and Rac1 (Fig 5A, panels i and j, and B) was similar (Fig 5A, panels e and f, and B). The number of total bacteria associated to HeLa cells did not change significantly (Fig 5C) indicating that the low percentage of internalized bacteria does not result from a defective bacterial adherence to cells. These results suggest that there is no additive effect of RhoA and Rac1 on *C*. *burnetii* internalization and that these GTPases participate in two parallel pathways. However, this hypothesis should be confirmed.

### ROCK, an effector of RhoA, is involved in the internalization of *C*. *burnetii*

It is known that during cell adhesion and migration, RhoA regulates stress fiber formation and contraction through the ROCK-dependent phosphorylation of the myosin light chain [64,65]. ROCK has also been demonstrated to be involved in phagocytosis [28,29]. Taking into account the recruitment of RhoA at the membranous fraction in cells infected with *C*. *burnetii* (Fig 3A and 3B), the role of RhoA in *C*. *burnetii* internalization (Fig 4A and 4B), and that ROCK is a downstream effector of RhoA, we decided to assess if this kinase participates in the bacterium uptake. One strategy to assess this issue was to diminish the cell synthesis of ROCK. To this end, HeLa cells were transfected with siRNA against ROCK and then infected for 4 h at 37°C. Cells were either lysed to analyze the depletion levels of ROCK protein (see Fig 5D) or fixed and processed as outlined above to evaluate the number of intracellular bacteria. The number of intracellular bacteria (red pseudo color, yellow arrowheads) was lower in HeLa cells transfected with ROCK siRNAs (Fig 5A, panels g and h, and B) in relation to the number of bacteria observed in cells transfected with the scramble siRNA (Fig 5A, panels a and b, and B). The depletion of the endogenous ROCK protein decreased *C*. *burnetti* internalization without significantly affecting the number of total bacteria associated to cells. This evidences that the inhibition of the bacterium internalization is not due to a defect in bacterial cell adherence (Fig 5C). These results suggest that ROCK plays a key role in the internalization of *C*. *burnetii*.

**Fig 6:**
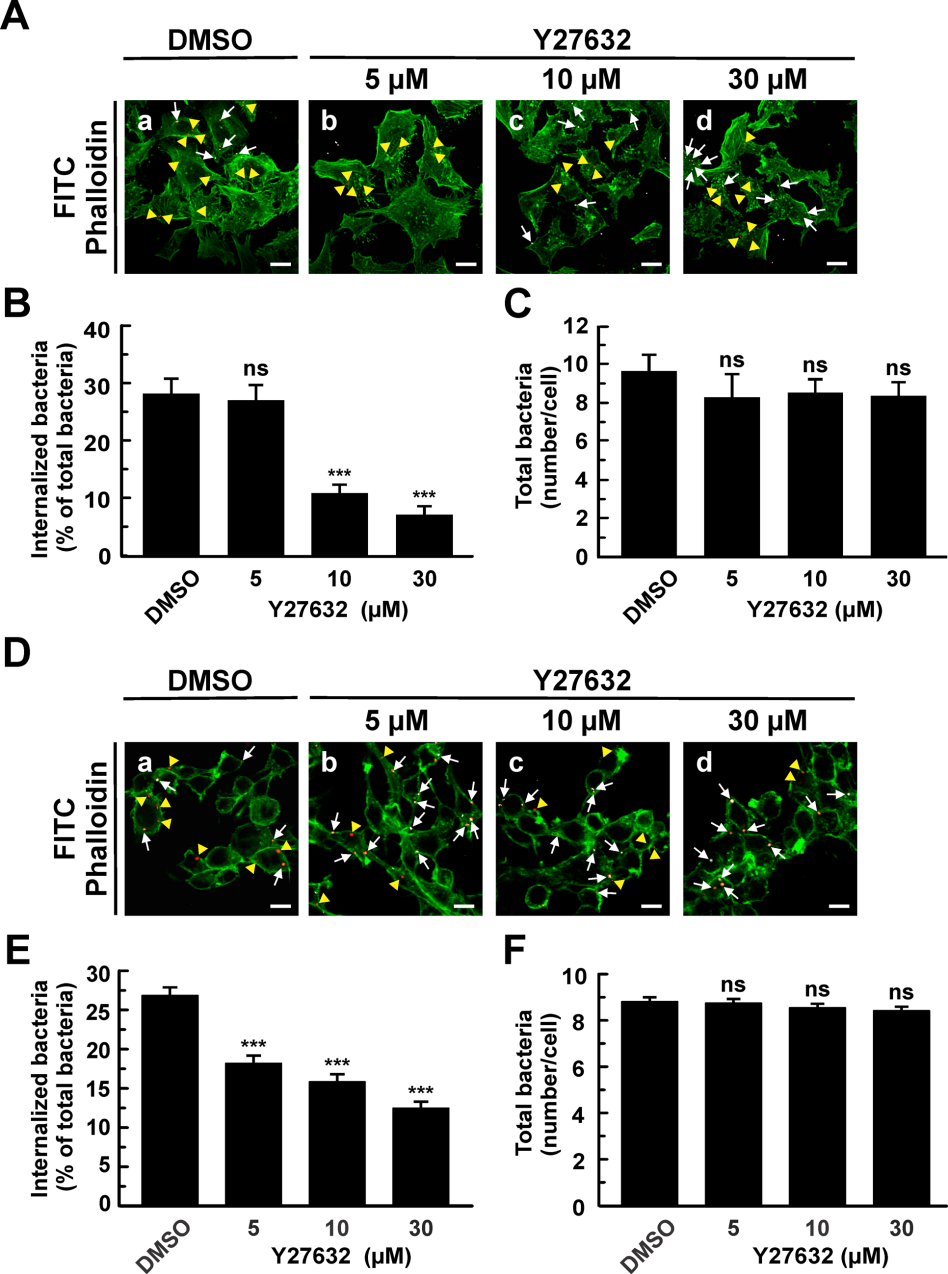
The specific inhibitor of ROCK, Y27632, diminishes *C*. *burnetii* internalization by HeLa or RAW cells. (A) HeLa or (D) RAW cells were infected with *C*. *burnetii* for 4 h at 37°C in the presence of 0.05% DMSO (control, A, panel a; D, panel a) or different concentrations of Y27632 (A, panels b-d; D, panels b-d). Cells were fixed and processed for indirect immunofluorescence to determine *C*. *burnetii* internalization and F-actin distribution as described in Materials and Methods. Cells were analyzed by confocal microscopy. Representative micrographs of cells are presented. F-actin was labeled with phalloidin-FITC (green). Representative micrographs of cells are presented. As indicated in Fig 1, in the merged images (A, panels a, b, c, and d; D, panels a, b, c, and d), extracellular *C*. *burnetii* is shown in white and red pseudo colors (arrows), while intracellular *C*. *burnetii* is shown in red pseudo color (yellow arrowheads). Between 100 and 120 cells and between 1200 and 1600 bacteria were counted in each experiment. Scale bar: 5 μm (A); 10 μm (D). Quantification of *C*. *burnetii* internalized by Y27632-treated HeLa (B) or RAW (E) cells. Quantification of total *C*. *burnetii* associated to HeLa (C) or RAW (F) cells. Results are expressed as means ± SE of three independent experiments. ***p < 0.001, compared to DMSO treatment (one-way ANOVA and Dunnett's *post hoc* test). ns: non-significant differences between groups (p > 0.05).

The other experimental approach to assess the role of ROCK in the uptake of *C*. *burnetii* was to inhibit the kinase by a specific inhibitor. To this end, HeLa or RAW cells were pre-incubated with different concentrations of Y27632, a ROCK inhibitor, and then infected for 4 h at 37°C. Cells were fixed and processed as described earlier so as to evaluate the number of intracellular bacteria. As displayed in Fig 6A and 6B, Y27632 inhibited *C*. *burnetii* internalization in a dose-dependent manner without affecting the total bacteria associated to the cells (Fig 6C), thus indicating that the adhesion of the bacteria to the cell surface is not affected by the inhibitor. As expected, the inhibitor altered the stress fibers formation (Fig 6A). Similar results were obtained using RAW cells (Fig 6D–6F). These results suggest that ROCK participates in the internalization of *C*. *burnetii* regardless of the cell lines used (e.g. epithelial cells or macrophages).

### The factor mDia1, an effector of RhoA, is recruited to the membrane fraction obtained from HeLa cells infected with *C*. *burnetii*

**Fig 7:**
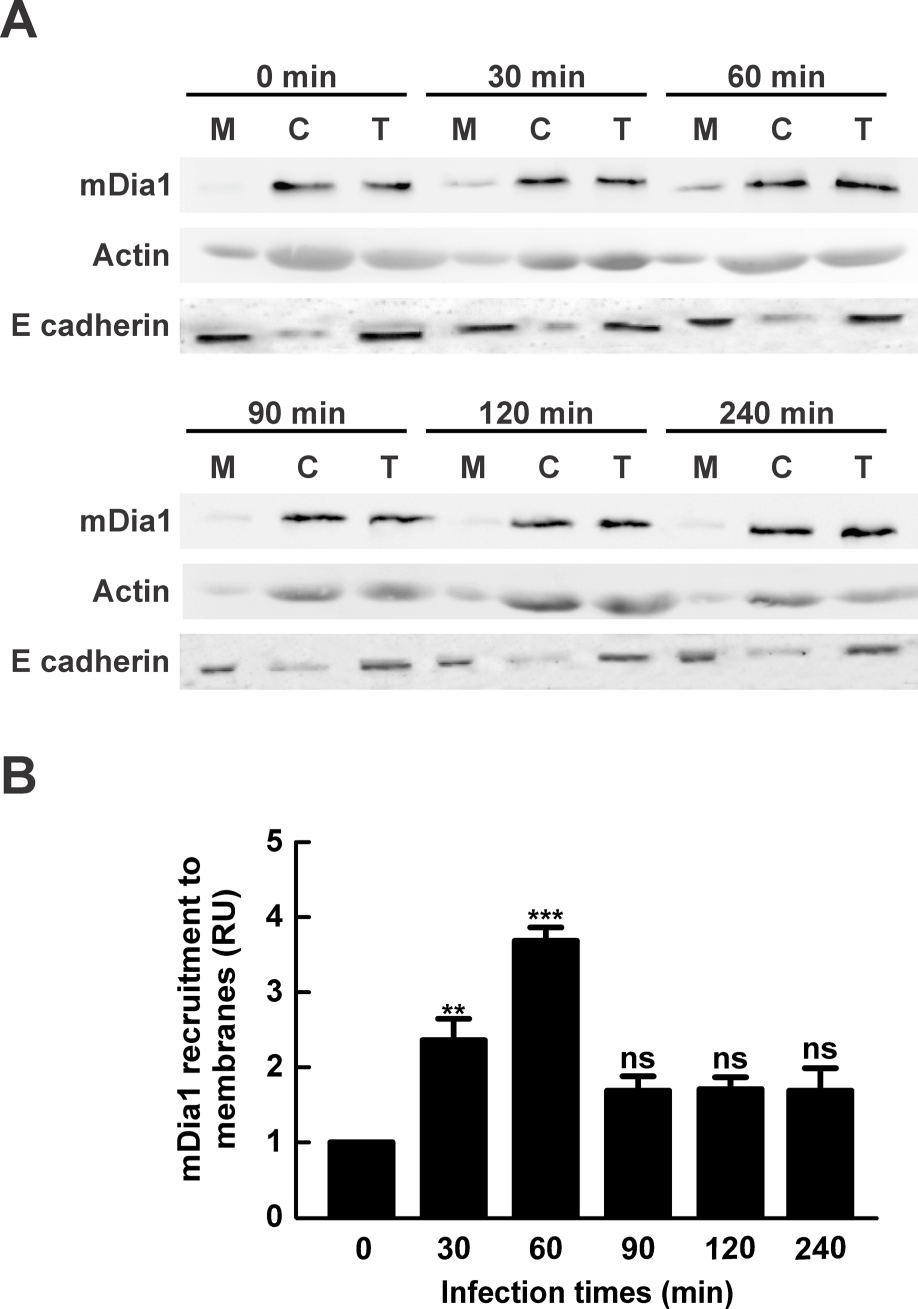
The factor mDia1 is recruited to membrane fraction obtained from cells infected with *C*. *burnetii*. (A) HeLa cells were infected with *C*. *burnetii* for different lengths of time, lysed and centrifuged to obtain postnuclear supernatant, membrane and cytosolic fractions. (A) Postnuclear supernatant (T: total), cytosol (C) and membrane (M) fractions were analyzed by SDS-PAGE and western blot using an antibody against mDia1. Anti-actin and anti-E cadherin antibodies were used as loading controls. (B) Quantification of mDia1 recruitment to membrane fraction. The band intensity of mDia1, E cadherin and actin was measured by the ImageJ software, and band intensity ratio between mDia1 and E cadherin in the membrane fractions was calculated. Results are expressed as means ± SE from at least three independent experiments. Means were compared with the 0 min infection condition by Student’s *t* test for single group mean (**p < 0.01, ***p < 0.001). ns: non-significant differences between groups (p > 0.05). (RU): Relative Units.

The factor mDia1 belongs to the formin family of proteins which behave as actin nucleator factors regulating actin dynamics [14,15]. Upon activation by RhoA, mDia1 is recruited to cell membrane to become functional [22,66]. Therefore, we decided to assess whether mDia1 is recruited to the cell membrane during infection. Then, HeLa cells were infected for different periods of time, lysed and centrifuged to obtain a membrane and a cytosolic fraction. Postnuclear supernatant (T: total fraction), cytosol (C) and membrane (M) fraction were analyzed by SDS-PAGE and Western blot. Fig 7A and 7B, shows that mDia1 recruitment to the membrane fraction increased after 30 min and reached its peak after 60 min of infection, a time point after which the levels of protein association decreased to basal levels. The mDia1 showed similar membrane recruitment kinetics when the experiments were performed with heat-killed *C*. *burnetii* (S1 Fig). The latter result strongly suggests that mDia1 is activated during *C*. *burnetii*-host´s cell interaction. Interestingly, the infection time of maximum mDia1 recruitment was similar to that observed for RhoA (Fig 3A and 3B).

### Internalization of *C*. *burnetii* by HeLa cells is stimulated by overexpression of a constitutively active variant of mDia1

The best-studied formins are diaphanous-related formins (DRFs), which are direct effectors of the Rho GTPases family. DRF proteins, such as mDia, have the actin assembly activity in the C-terminal end and the regulatory region in the N-terminal end, which mediates intramolecular interactions with the C terminus to maintain formins in an autoinhibited state [67]. The C-terminal end contains three structural and functional elements: (*a*) the profilin-binding FH1, (*b*) the actin-binding FH2, and (*c*) the diaphanous autoregulatory domain (DAD). The N terminus consists of four distinct structural domains, including (*a*) the GTPase binding domain (GBD), which binds Rho family GTPases in the GTP-bound state; (*b*) the diaphanous inhibitory domain (DID), which binds the C-terminal autoinhibitory DAD segment and also interacts with Rho GTPases; (*c*) the dimerization domain (DD); and (*d*) a coiled-coil (CC) region. The inactive mDia adopts an autoinhibitory conformation mediated by an intramolecular interaction between the DAD, DID and a part of the GBD domains. Binding of GTP-bound Rho protein (GTP Rho) to the GBD domain promotes formin release from the autoinhibited state [67,68].

**Fig 8:**
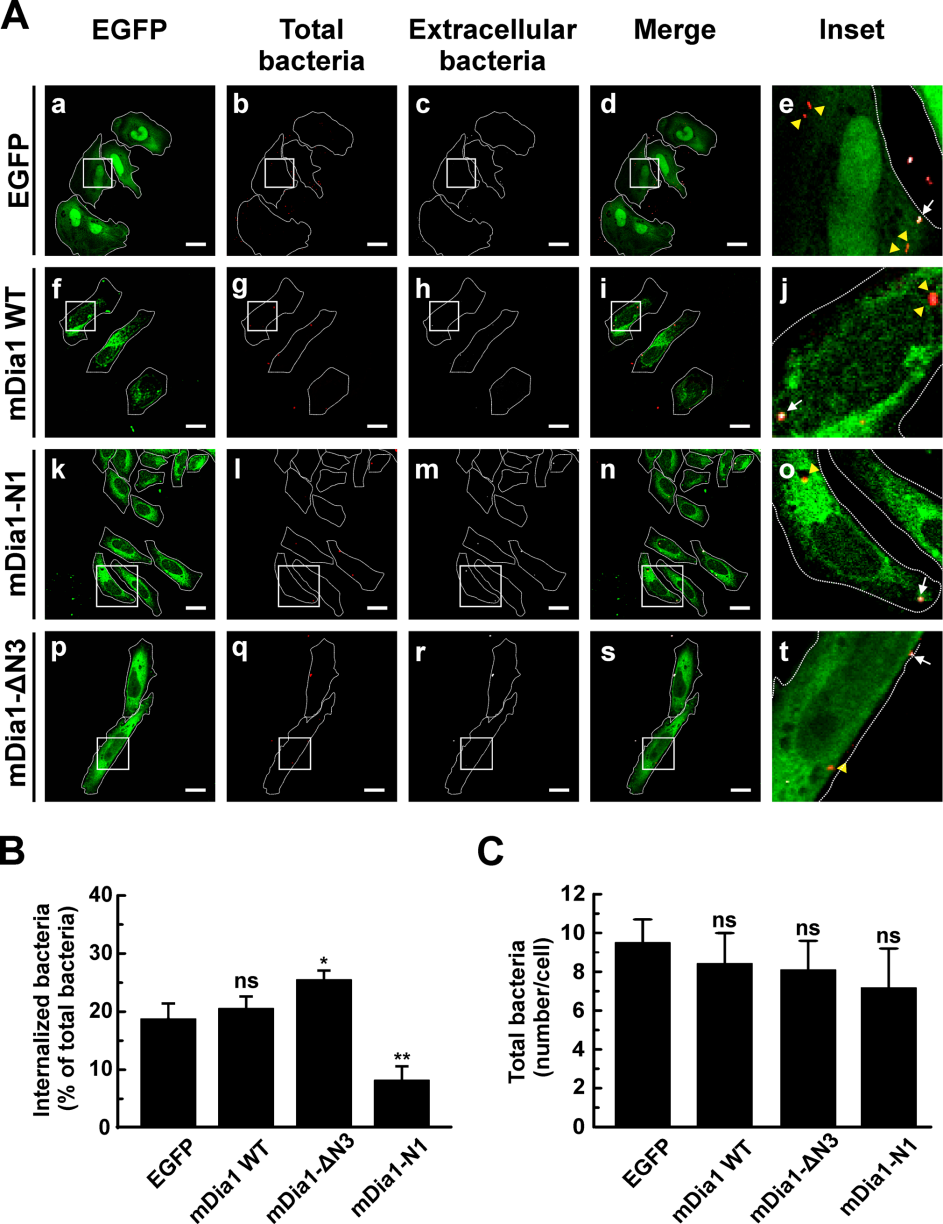
The overexpression of the dominant negative mutants of mDia1 inhibits internalization of C. *burnetii*. (A) HeLa cells were transfected with pEGFP (panels a-e), pEGFP-mDia1 WT (panels f-j), pEGFP-mDia1-N1 (dominant negative form) (panels k-o) or pEGFP-mDia1-ΔN3 (constitutively active form) (panels p-t). Transfected cells were infected for 4 h at 37°C with *C*. *burnetii*. Cells were fixed and processed for immunofluorescence to determine *C*. *burnetii* internalization as described in Materials and Methods. Cells were analyzed by confocal microscopy. Representative micrographs of cells are presented. As indicated in Fig 1, extracellular and total bacteria were stained in white pseudo color (panels c, h, m, and r) and red pseudo color (panels b, g, l, and q), respectively. In the merged images (panels d, i, n, and s) and the insets of merged images (panels e, j, o, and t), extracellular *C*. *burnetii* is shown in white and red pseudo colors (arrows), while intracellular *C*. *burnetii* is shown in red pseudo color (yellow arrowheads). Scale bar: 5 μm. (B) Quantification of *C*. *burnetii* internalized by transfected HeLa cells. (C) Quantification of total *C*. *burnetii* associated to HeLa cells. Between 40 and 60 cells and between 400 and 600 bacteria were counted in each experiment. Results are expressed as means ± SE of three independent experiments. *p < 0.05, **p < 0.01 compared to the EGFP control (one-way ANOVA and Dunnett's *post hoc* test). ns: non-significant differences between groups (p > 0.05).

To test whether mDia1 plays a role in the internalization process, HeLa cells were transfected with pEGFP-mDia1 wild type (WT), pEGFP-mDia1-N1 or pEGFP-mDia1-ΔN3. The truncated mutants mDia1-N1 (N-terminal Rho-binding domains) and mDia1-ΔN3 (C-terminal FH1 and FH2 domains) function as dominant negative and constitutively active forms, respectively [18]. After transfection, cells were infected for 4 h at 37°C, fixed and processed as specified above to evaluate the number of intracellular bacteria. The different overexpressed constructs of mDia1 (Fig 8A) featured a distribution similar to that reported by Watanabe et al [18]. *C*. *burnetii* internalization diminished in cell overexpressing EGFP-mDia1-N1 suggesting that the FH2 and FH3 domains are important for actin cytoskeleton remodeling involved in *C*. *burnetii* uptake. In turn, an increase in internalization was observed in cells overexpressing the active EGFP-mDia1-ΔN3 construct (Fig 8A and 8B). The overexpression of all constructs did not significantly affect the amount of total bacteria associated to the cells. This finding evidences that changes in the bacterium internalization process is not due to a defect in bacteria adherence to the cells (Fig 8C).

Taken together, these results indicate that the RhoA effector mDia1 is activated and recruited to the membranes upon infection with *Coxiella* and, in addition, its function is important for *C*. *burnetii* entry into host cells.

### The overexpression of a constitutively active variant of mDia1 stimulated internalization of *C*. *burnetii* in RhoA-depleted HeLa cells

**Fig 9:**
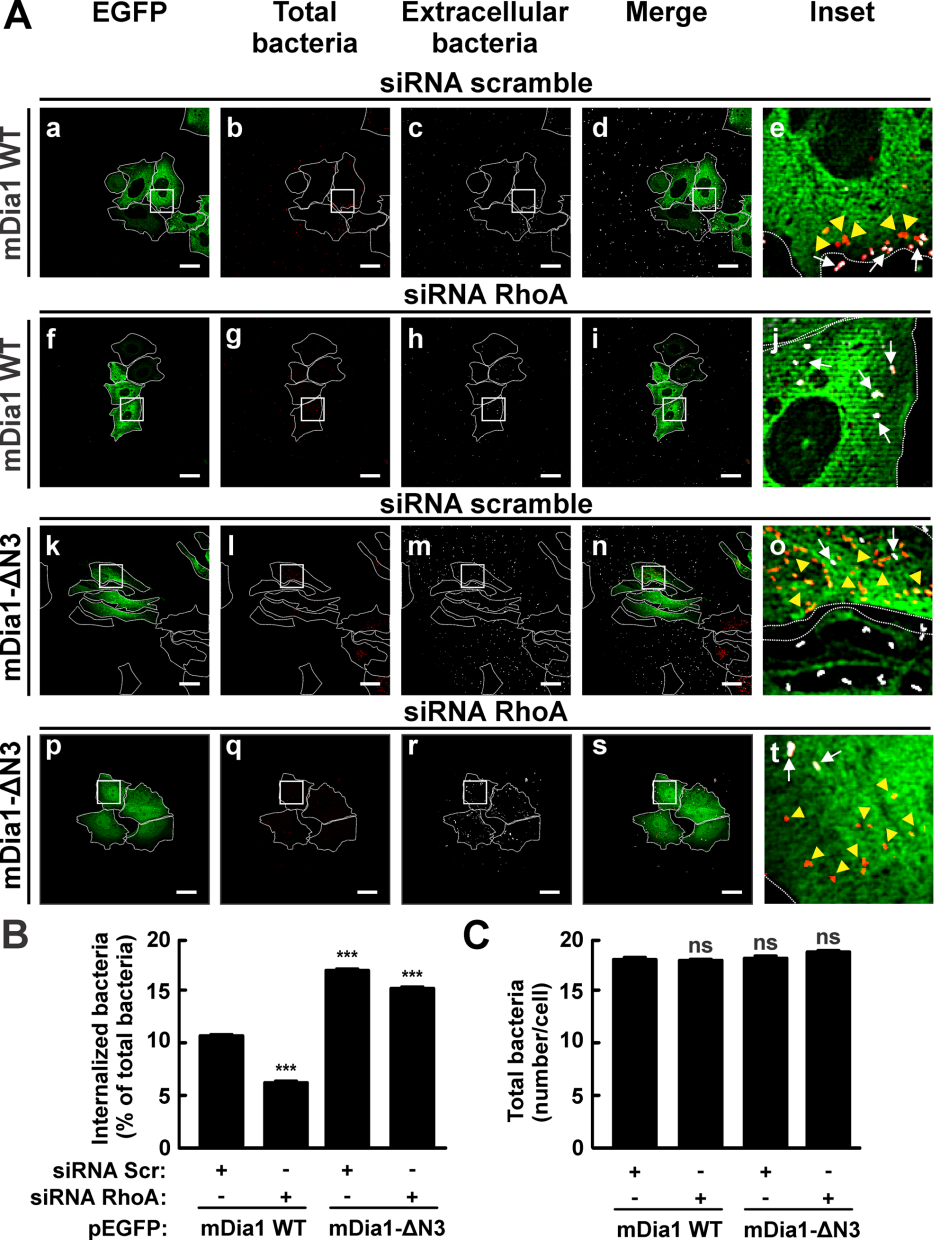
The overexpression of the constitutively active form of mDia1 restored the entry of *C*. *burnetii* into RhoA-knocked down HeLa cells. (A) HeLa cells were cotransfected with pEGFP-mDia1 WT (panels a-j) or pEGFP-mDia1-ΔN3 (constitutively active form) (panels k-t) and scramble siRNA (panels a-e) or RhoA siRNA (panels p-t). Transfected cells were infected for 4 h at 37°C with *C*. *burnetii*. Cells were fixed and processed for immunofluorescence to determine *C*. *burnetii* internalization as described in Materials and Methods. Cells were analyzed by confocal microscopy. Representative micrographs of cells are presented. As indicated in Fig 1, extracellular and total bacteria were stained in white pseudo color (panels c, h, m, and r) and red pseudo color (panels b, g, l, and q), respectively. In the merged images (panels d, i, n, and s) and the insets of merged images (panels e, j, o, and t), extracellular *C*. *burnetii* is shown in white and red pseudo colors (arrows), while intracellular *C*. *burnetii* is shown in red pseudo color (yellow arrowheads). Scale bar: 5 μm. (B) Quantification of *C*. *burnetii* internalized by cotransfected HeLa cells. (C) Quantification of total *C*. *burnetii* associated to HeLa cells. Between 40 and 60 cells and 400 and 600 bacteria were counted in each experiment. Results are expressed as means ± SE of three independent experiments. ***p < 0.001 compared to the EGFP control (one-way ANOVA and Dunnett's *post hoc* test). ns: non-significant differences between groups (p > 0.05). Scr: scramble siRNA.

To examine the relationship between mDia91 and RhoA in the internalization process, HeLa cells were cotransfected with siRNAs targeted to RhoA and pEGFP-mDia1 WT or pEGFP-mDia1-ΔN3 (constitutively active form) and then infected for 4 h at 37°C. Cells were fixed and processed as specified above to evaluate the number of intracellular bacteria. The internalization of *C*. *burnetii* was found to be diminished in WT EGFP-mDia1 overexpressing cells that were transfected with RhoA siRNA as compared to those transfected with scramble siRNA. Interestingly, the internalization of *C*. *burnetii* increased in EGFP-mDia1-ΔN3 overexpressing cells that were transfected with RhoA or scramble siRNAs (Fig 9A and 9B). These results indicate that the constitutively active form of mDia1 restores *C*. *burnetii* uptake in cells depleted of RhoA. As expected, once mDia1 is activated, its function can be performed independently of RhoA.

## Discussion

Several pathogens are able to modulate the host´s cells functions as an evasion mechanism. During the interaction with host´s cells, the microorganisms can remain adhered to the cell surface or being internalized and then become sequestered into an intracellular vacuole that prevents the fusion with lysosomes. Once internalized, other pathogens lyse the phagosome and multiply within the cytoplasm using host’s actin filaments to disseminate to neighboring cells [69].

It is well known that *C*. *burnetii* is a pathogen that transits along the phagocytic pathway interacting with different endosomal compartments, generating a PV with autophagolysosomal characteristics; however, the *C*. *burnetii* entry to the host´s cells is a poorly characterized mechanism. Nonetheless, it has previously been demonstrated that *C*. *burnetii* NMII and NMI internalization is diminished in fibroblasts and monocytes treated with cytochalasin D, an inhibitor of actin polymerization, suggesting a participation of the actin cytoskeleton in that process [53–55]. In this work, similar results were obtained in HeLa and RAW cells infected with *C*. *burnetii* NMII and treated with cytochalasin D (data not shown). Meconi et al. [56] have postulated that monocytes incubated with *C*. *burnetii* NMI exhibited dramatic plasma membrane extensions and protrusions associated to actin cytoskeleton reorganization. Conversely, only a few membrane folds were observed in cells incubated with the attenuated *C*. *burnetii* NMII [56]. However, the molecular events related to the morphological changes observed in the plasma membrane and the entry of *C*. *burnetii* NMI or NMII into monocytes were not analyzed. Interestingly, and despite the more profound changes in the host´s plasma membrane induced by virulent *C*. *burnetii* compared to that caused by the avirulent bacterium, the internalization of the latter was more efficient [54]. All together, these observations are in agreement with our experimental model in which discreet plasma membrane extensions stimulated by avirulent *C*. *burnetti* are associated with a minor actin cytoskeleton rearrangement that allows effective *C*. *burnetii* internalization. Adhesion and invasion of bacteria to the host’s cell can be either an active or a passive process. The active one, termed "triggering mechanism", involves a bacterial type 3 secretion system (T3SS) that injects effectors into the host´s cell cytoplasm to stimulate uncontrolled actin rearrangement ruffles formation and bacteria internalization. The passive process or "zippering mechanism", involves a narrow interaction between bacteria (ligands) and host´s cell (receptors) surfaces, pseudopodia formation and bacterial uptake [70,71]. Cumulative evidence indicates that *C*. *burnetii* uses a zipper mechanism to entry into the host´s cell [72]. *C*. *burnetii* enters the cell by a sinking process that involves the extension of much lower prominent pseudopodia as compared to other bacteria or IgG-opsonized particles [73,74].

We have previously demonstrated that the formation of *C*. *burnetii* containing PV depends on actin and GTPases of the Rho family [51]. More recently, after studying the early interaction between *C*. *burnetii* and the host´s cell, we showed that cortactin, a protein that participates in the regulation of actin cytoskeleton dynamics, plays a role in the *C*. *burnetii* internalization step [52]. The present study contributes to further understand the role of the GTPases of the Rho family in bacterial pathogenesis. We provide evidence that Rac1, Cdc42 and, particularly, RhoA, and their effectors mDia1 and ROCK, are involved in signal transduction pathways that are involved in the internalization of avirulent *C*. *burnetii* into phagocytic and non-phagocytic cells. The use of different cellular models have facilitated important progresses to better understand *C*. *burnetii*-host´s cells interaction [75–77], as well as to study the host-cell interplay of other pathogens [78,79]. In this work, RAW and HeLa cell lines were used as professional and non-professional phagocytes, respectively, demonstrating that similar molecular mechanisms are involved in both infection models.

The GTPases of the Rho family, main regulators of actin cytoskeleton dynamics, participate in phagocytosis and invasion of several pathogens [80,81]. Our data demonstrate the role of Rho GTPases in *C*. *burnetii* internalization in both HeLa and RAW cells by using *C*. *difficile* toxin B, a pharmacological tool used in other experimental models of infection of HeLa cells with *Chlamydia trachomatis* [82] or *Neisseria meningitidis* [83]. Meconi et al [56] have demonstrated that monocytes treated with the C3 exotransferase of *C*. *botulinum*, an inhibitor of Rho GTPases, drastically diminished the formation of membrane protrusions induced by infection with *C*. *burnetii* NMI [56]; however, these authors have not assessed the bacterium internalization. In this study, *C*. *difficile* toxin B abrogated *C*. *burnetii* uptake in HeLa as well as RAW cells, suggesting that the GTPases of the Rho family have a critical regulatory function in the internalization process.

It has previously been demonstrated that GTPases, when activated, are recruited to membranes from the cytoplasm [62,63,84]. We observed that Rac1 and RhoA associated to a membrane fraction obtained from cells infected with *C*. *burnetii* after 30 and 60 min, respectively. This result suggests that the bacterium sequentially activates different members of the Rho GTPases family during infection. By the FRET technique, it has been shown that during IgG-opsonized erythrocytes phagocytosis, Rac1 and Rac2 were activated shortly after Cdc42 activation [37]. More interestingly, during phagocytosis of complement-opsonized zymosan particles, RhoA activation was detected by western blotting after 20 min of internalization [39]. This activation time is similar to the one observed in our *C*. *burnetii* infection model. Unfortunately, the antibody against Cdc42 used was unable to detect the protein even in the postnuclear supernatant. Thus, the activation of Cdc42 during *C*. *burnetii* infection could not be ruled out.

Interestingly, it has been reported that the activation level of Rho GTPases in cell lines, even in cells strongly stimulated, is often very low, around 5% of the total GTPase pool, to be easily detected by techniques such as western blot [85–88]. Moreover, the cycling of GTPases between membrane and cytoplasm, and their interaction with RhoGDI affects the sensibility of immunostaining experiments as well as GFP-Rho overexpression experiments because a very low fraction of GTPase is activated in a spatio-temporal way in the cell [87,89]. The low recruitment to the membrane and the fast cycling of the Rho proteins may explain the fact that we could not detect the EGFP-mDia1 at the *Coxiella* entry sites by fluorescent microscopy. Biosensors have been used to visualize, at high resolution, the activation of Rho GTPases in living cells [86,87,90]. The application of these tools in future studies will greatly enhance the ability to analyze and understand the role of GTPase activation during *C*. *burnetii* infection.

In the present study, we demonstrated the importance of the active state of Rho GTPases in *C*. *burnetii* internalization by using HeLa cells overexpressing dominant negative mutants which significantly decreased the bacterium entry into the cells. A similar experimental approach has been used to evidence the internalization process of other pathogens. *Chlamydia caviae* internalization is controlled by Cdc42 and Rac1 [80], whereas in the uptake of *Chlamydia trachomatis*, only Rac1 is involved [82]. These results suggest that the process is highly dependent on bacterium species. However, in some cases, the relationship between uptake and Rho proteins seems to be related to the type of host´s cells. For instance, *Listeria* entry is Rac-dependent in Vero cells but requires both Rac1 and Cdc42 in Ref52 fibroblasts [91] or mainly Cdc42 in HeLa cells [92]. The internalization of *Neisseria meningitidis* into COS epithelial cells expressing specific Opa-receptors is mediated by Cdc42 and Rac1, but is independent of RhoA activity [83]. Interestingly, we show herein that the three GTPases are involved in *C*. *burnetii* internalization by HeLa cells, thus suggesting that these GTPases may act synergistically during the entry of these bacteria. Burnham et al [93] have reported comparable results during invasion of HeLa cells by *Streptococcus*. The role of Rho GTPases has also been tested by using specific siRNAs. Our results demonstrate that the level of *C*. *burnetii* uptake in cells depleted of RhoA or Rac1 was similar to that observed in cells depleted of the two GTPases. The double-knockdown results suggest that there exists no additive effect between RhoA and Rac1 and that these GTPases participate in two parallel pathways. We believe that these hypotheses remain to be tested, which is difficult to attain, considering the complex crosstalk between the GTPases of the Rho family. It is known that the crosstalk between Rho-GTPase signals that involves formation of complexes between regulators of the same and different GTPases (GAP, GEF, membrane receptors, downstream effectors) hinders the interpretation of physiological cell processes [87,94].

It is noteworthy that the recruitment of mDia1, an effector of RhoA, to membrane fractions of cells infected with *C*. *burnetii* suggests that mDia1 acquires an open conformation that exposes the actin nucleation sites, which allows the actin polymerization needed for *C*. *burnetii* internalization. This observation is consistent with the stimulation of *C*. *burnetii* uptake in cells overexpressing the mDia1 positive mutant ΔN3, which contains the actin nucleation domain, and also with the inhibition of the uptake in cells overexpressing the negative mutant N1 that only contains the Rho binding domain. Other models have shown that this mutant stimulates cell elongation and the formation of parallel thin actin cables, while the negative truncated mutant inhibits actin-fiber formation by sequestering active Rho proteins [18,95]. Thus, it is likely that a similar mechanism is working for *Coxiella* infection. The participation of mDia1 and related proteins has also been observed in the infection process of other bacterial pathogens. *Shigella flexneri* and *Rickettsia rickettsii* utilize mDia1 and Sca2 (formin mimic protein), respectively, to induce actin polymerization and, therefore, intracellular motility and spreading [96,97]. During infection with *Vibrio cholerae*, VopF, a TTSS effector with formin-like activity, participates *in vivo* in intestinal colonization and, *in vitro*, in alterations of actin cytoskeleton and cell morphology in a manner similar to formin [98]. Recently, the role of formin FHOD1 in *S*. *typhimurium* entry into HeLa cell has been demonstrated [99].

It has also been shown that mDia1 is recruited to endosomes in HeLa cells suggesting its role in controlling endosomal trafficking [95,100]. Colucci-Guyon et al [21] observed that mDia1, together with actin, was recruited early to the phagocytic cup during CR3-mediated phagocytosis in RAW264.7 macrophages. Interestingly, *C*. *burnetii* interacts with CR3 and the αVβ3 integrin of the host’s cells [54]. Therefore, it is tempting to hypothesize that mDia1 may regulate *C*. *burnetii* internalization through CR3. Further studies should be conducted to assess this hypothesis.

In this work we have provided evidence that the kinase ROCK is also a key player in *C*. *burnetii* internalization, since the process was hampered in cells treated with a ROCK inhibitor, or by silencing the protein with a specific siRNA. These observations are in agreement with the role of ROCK in CR3-mediated phagocytosis in J774.A1 and RAW264.7 macrophages and in Cos-7 fibroblasts [21,28]. Likewise, the *Salmonella* invasion of non-phagocytic cells was significantly decreased by the ROCK inhibitor Y27632 [101]. More recently, Truong et al [99] have demonstrated the requirement of ROCK II, but not of ROCK I, in *S*. *typhimurium* uptake into HeLa cells using specific siRNAs. In our model, employing a similar experimental approach, we demonstrated the role of ROCK I, yet the participation of ROCK II cannot be ruled out if the effect of the general ROCK inhibitor is considered. ROCK has also been involved in the infection of other pathogens such as *E*. *coli* K1 [102] that invades brain microvascular endothelial cells, and EHV-1 (equine herpes virus type 1) in a CHO-K1 cell line [103]. Thus, it is evident that ROCK participates in infection processes involving different types of microorganisms comprising not only bacteria but also viruses.

In conclusion, our results indicate that the active forms of RhoA, Cdc42 and Rac1 play an important role and work sequentially in the entry of *C*. *burnetii* into the host´s cells, regulating the actin rearrangement needed for this process. It could be speculated that these GTPases may work together in a cooperative manner, but this hypothesis needs to be further tested. Moreover, we report for the first time, that the RhoA effectors mDia1 and ROCK are involved in a signal transduction mechanism that favors *C*. *burnetii* uptake, highlighting the importance of these molecules in *Coxiella* entry into host´s cells.

## Supporting Information

S1 Fig RhoA, Rac1 and mDia1 are recruited to the membrane fraction obtained from HeLa cells infected with heat-killed *C*. *burnetii*. HeLa cells were infected with heat-killed *C*. *burnetii* for different lengths of time, lysed and centrifuged to obtain postnuclear supernatant, membrane and cytosolic fractions as described in Materials and Methods. (A) Postnuclear supernatant (T: total), cytosol (C) and membrane (M) fractions were analyzed by SDS-PAGE and Western blot using antibodies against RhoA, Rac1 and mDia1. Anti-actin and anti-E cadherin antibodies were used as loading controls. (B) Quantification of RhoA, Rac1 and mDia1 recruitment to the membrane fraction. The band intensity of RhoA, Rac1, mDia1, E cadherin and actin was measured by the ImageJ software, and band intensity ratio between RhoA and E cadherin, Rac1 and E cadherin, and mDia1 and E cadherin in the membrane fractions was calculated. Results are expressed as means ± SE from at least three independent experiments. Means were compared with the 0 min infection condition by Student’s *t* test for single group mean (*p < 0.05, ***p < 0.001). ns: indicates non-significant differences between groups (p > 0.05). (RU): Relative Units.
(TIF)
