# Supplementary figures and images for: Coxiella burnetii Phagocytosis Is Regulated by GTPases of the Rho Family and the RhoA Effectors mDia1 and ROCK
Source: PLoS One. 2015 Dec 16;10(12):e0145211. doi: 10.1371/journal.pone.0145211 (PMC4682630; doi:10.1371/journal.pone.0145211)

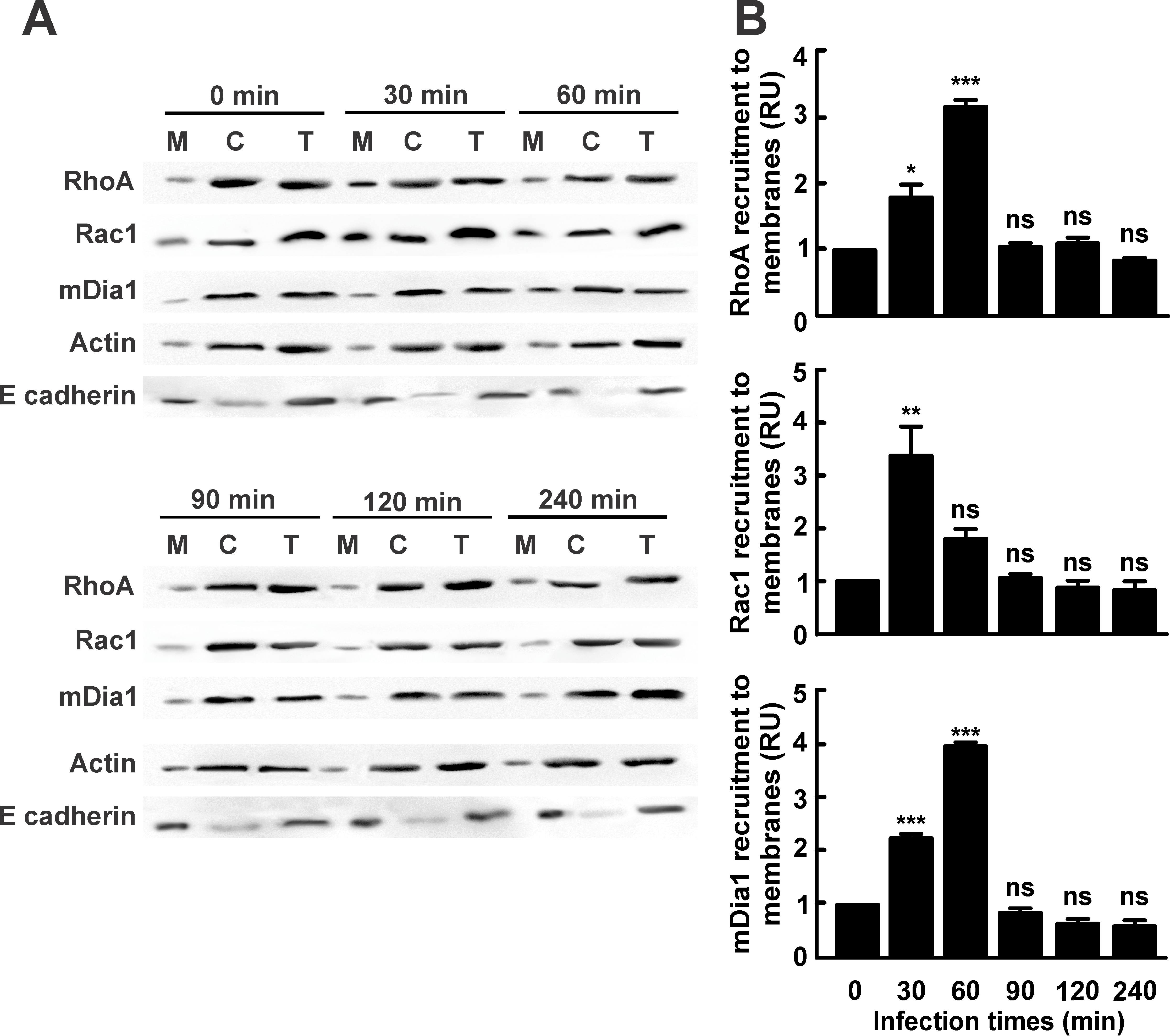

Supplement: S1 Fig — HeLa cells were infected with heat-killed C. burnetii for different lengths of time, lysed and centrifuged to obtain postnuclear supernatant, membrane and cytosolic fractions as described in Materials and Methods. (A) Postnuclear supernatant (T: total), cytosol (C) and membrane (M) fractions were analyzed by SDS-PAGE and Western blot using antibodies against RhoA, Rac1 and mDia1. Anti-actin and anti-E cadherin antibodies were used as loading controls. (B) Quantification of RhoA, Rac1 and mDia1 recruitment to the membrane fraction. The band intensity of RhoA, Rac1, mDia1, E cadherin and actin was measured by the ImageJ software, and band intensity ratio between RhoA and E cadherin, Rac1 and E cadherin, and mDia1 and E cadherin in the membrane fractions was calculated. Results are expressed as means ± SE from at least three independent experiments. Means were compared with the 0 min infection condition by Student’s t test for single group mean (*p < 0.05, ***p < 0.001). ns: indicates non-significant differences between groups (p > 0.05). (RU): Relative Units. (TIF) [file pone.0145211.s001.tif]
